# Rituximab retention rate in systemic sclerosis: a long term real-life multicentre study

**DOI:** 10.1093/rheumatology/keae280

**Published:** 2024-05-15

**Authors:** Giacomo De Luca, Enrico De Lorenzis, Corrado Campochiaro, Fabio Cacciapaglia, Nicoletta Del Papa, Elisabetta Zanatta, Paolo Airò, Maria Grazia Lazzaroni, Dilia Giuggioli, Maria De Santis, Gabriella Alonzi, Stefano Stano, Marco Binda, Beatrice Moccaldi, Antonio Tonutti, Silvia Cavalli, Veronica Batani, Gerlando Natalello, Florenzo Iannone, Maria Antonietta D’Agostino, Lorenzo Dagna, Marco Matucci-Cerinic, Silvia Laura Bosello

**Affiliations:** Scleroderma Unit, Unit of Immunology, Rheumatology, Allergy and Rare Diseases, IRCCS San Raffaele Hospital, Milan, Italy; Vita-Salute San Raffaele University, Milan, Italy; Rheumatology Unit, Università Cattolica del Sacro Cuore, Fondazione Policlinico Universitario A. Gemelli—IRCCS, Rome, Italy; Scleroderma Unit, Unit of Immunology, Rheumatology, Allergy and Rare Diseases, IRCCS San Raffaele Hospital, Milan, Italy; Vita-Salute San Raffaele University, Milan, Italy; Rheumatology Unit, DiMePRe-J University of Bari, Bari, Italy; Scleroderma Clinic, ASST Gaetano Pini-CTO, Milan, Italy; Rheumatology Unit, Padova University Hospital, Padova, Italy; Scleroderma Unit, UOC Rheumatology and Clinical Immunology, ASST Spedali Civili, Brescia, Italy; Scleroderma Unit, UOC Rheumatology and Clinical Immunology, ASST Spedali Civili, Brescia, Italy; Rheumatology Unit, School of Medicine, University of Modena and Reggio Emilia, Modena; Rheumatology and Clinical Immunology, IRCCS Humanitas Research Hospital, Rozzano, Italy; Department of Biomedical Sciences, Humanitas University, Pieve Emanuele, Italy; Rheumatology Unit, Università Cattolica del Sacro Cuore, Fondazione Policlinico Universitario A. Gemelli—IRCCS, Rome, Italy; Rheumatology Unit, DiMePRe-J University of Bari, Bari, Italy; Rheumatology Unit, Padova University Hospital, Padova, Italy; Rheumatology Unit, Padova University Hospital, Padova, Italy; Rheumatology and Clinical Immunology, IRCCS Humanitas Research Hospital, Rozzano, Italy; Department of Biomedical Sciences, Humanitas University, Pieve Emanuele, Italy; Scleroderma Clinic, ASST Gaetano Pini-CTO, Milan, Italy; Scleroderma Unit, Unit of Immunology, Rheumatology, Allergy and Rare Diseases, IRCCS San Raffaele Hospital, Milan, Italy; Vita-Salute San Raffaele University, Milan, Italy; Rheumatology Unit, Università Cattolica del Sacro Cuore, Fondazione Policlinico Universitario A. Gemelli—IRCCS, Rome, Italy; Rheumatology Unit, DiMePRe-J University of Bari, Bari, Italy; Rheumatology Unit, Università Cattolica del Sacro Cuore, Fondazione Policlinico Universitario A. Gemelli—IRCCS, Rome, Italy; Scleroderma Unit, Unit of Immunology, Rheumatology, Allergy and Rare Diseases, IRCCS San Raffaele Hospital, Milan, Italy; Vita-Salute San Raffaele University, Milan, Italy; Scleroderma Unit, Unit of Immunology, Rheumatology, Allergy and Rare Diseases, IRCCS San Raffaele Hospital, Milan, Italy; Vita-Salute San Raffaele University, Milan, Italy; Rheumatology Unit, Università Cattolica del Sacro Cuore, Fondazione Policlinico Universitario A. Gemelli—IRCCS, Rome, Italy

**Keywords:** systemic sclerosis, rituximab, retention rate, real-life data, B-cells

## Abstract

**Objectives:**

To report real-life data on rituximab retention rate as an indicator of safety and efficacy in a multicentric national cohort of systemic sclerosis patients.

**Methods:**

SSc patients treated with rituximab and followed for at least 36 months were included, clinically characterized and longitudinally monitored. A competing risk analysis with sub-hazard ratio (sHR) definition was performed to explore the clinical variables linked to specific cause of rituximab discontinuation.

**Results:**

One-hundred and fifty-two SSc-patients [mean age 47.3 (12.3) years; females 79.6%; diffuse disease 77.6%; anti-topoisomerase-I positivity 63.2%] were evaluated over a median (interquartile range) time of 3.3 (1.7–5.0) years. The primary indications for rituximab were interstitial lung disease (38.8%), worsening skin fibrosis (36.8%) and arthritis (13.8%); 138 patients (90.8%) received more than one rituximab course. The 5-year rituximab retention rate was 59.9% (44.6–64.7%). Clinical response was the most common reason for rituximab discontinuation [5.7; 95% CI: (3.7–8.4) per 100 patient-years] and was associated with a shorter disease duration (sHR 0.8; 95% CI: 0.7, 0.9), anti-topoisomerase-I negativity (sHR 0.4; 95% CI: 0.2, 0.9), previous digital ulcers (sHR 2.6; 95% CI: 1.1, 6.2) and no history of arthritis (sHR 0.3; 95% CI: 0.1, 0.8). Treatment failure was the second cause of rituximab discontinuation [3.7 (95% CI: 2.2, 6.0) per 100 patient-years] and was associated with anti-centromere antibody positivity (sHR 2.8; 95% CI: 1.1, 7.4) and anti-topoisomerase-I negativity (sHR 0.2; 95% CI: 0.1, 0.6). Adverse events (AEs) were the less common cause of discontinuation [3.1 (95% CI: 1.7, 5.2) per 100 patient-years], associated with limited cutaneous subset (sHR 3.4; 95% CI: 1.2, 9.7) and previous mycophenolate mofetil treatment (sHR 4.5; 95% CI: 1.2, 16.3).

**Conclusion:**

Rituximab is a safe and effective treatment in SSc: clinical response emerged as the primary reason for rituximab discontinuation, and AEs had a limited impact on treatment persistence. The identification of specific disease features associated with a response to rituximab will be useful in the management of SSc-patients.

Rheumatology key messages

- Rituximab is safe in systemic sclerosis: adverse events have a limited impact on treatment persistence.
- Rituximab is effective in systemic sclerosis, clinical response being the primary reason for discontinuation.
- The identification of specific disease features associated with a response to rituximab is eagerly awaited.

## Introduction

Systemic sclerosis (SSc) is a severe immune-mediated disease characterized by diffuse vascular damage and aberrant activation of the immune system, resulting in inflammation and fibrosis of skin and internal organs [1, 2]. Immunosuppressive strategies to treat SSc are not yet established as only a limited number of treatments have shown efficacy and safety [3–7]. Consequently, the disease-related mortality rate remains high, primarily due to lung and heart involvement [8].

In SSc patients with progressive skin and lung involvement, experimental and clinical data support the therapeutic use of the monoclonal antibody rituximab (RTX), which targets human CD20 [4, 5, 7, 9–11]. The recent large prospective non-randomized EUSTAR study confirmed the effectiveness and safety of RTX, showing significant improvement in skin fibrosis and a stabilization of lung function [9].

Recently, in the DESIRES study (a double-blind, investigator-initiated, randomized, placebo-controlled phase 2 clinical trial) RTX led to significant reduction of skin involvement extension, evaluated by the modified Rodnan skin score (mRSS) [7] compared with placebo at 24 weeks. Furthermore, the study indicated that in patients with SSc-related interstitial lung disease (ILD), the change in the percentage of predicted forced vital capacity (FVC) favoured the RTX-treated group over placebo. Subsequently, these findings were validated in a 24-week open-label extension phase of the DESIRES trial [12]. The usefulness of RTX to treat SSc-related ILD also emerged in the recent RECITAL study, a double-blind, double-dummy, randomized, controlled, phase 2b trial showing that RTX is not superior to cyclophosphamide to treat patients with ILD associated with connective tissue diseases, including SSc [13]. Participants in both groups, indeed, had increased FVC at 24 months and improved quality of life. Nevertheless, the long-term efficacy and safety of RTX therapy in SSc have not been fully confirmed in prospective clinical trials. Likewise, real-life data on the long-term safety and efficacy of RTX are limited.

The aim of this study was to evaluate the real-life RTX retention rate as an indicator of safety and efficacy in a multicentric cohort of Italian SSc patients.

## Methods

### Study design, sample and data collection

An observational retrospective longitudinal study was designed to analyse data from SSc patients followed up in eight Italian Scleroderma referral centres.

The patients included in the analysis, classified as SSc according to SSc 2013 ACR/EULAR classification criteria [14], were treated with at least one complete RTX course between October 2006 and February 2020, and had a follow-up of at least 36 months. The study included patients treated with RTX originator (RTX-O) or RTX biosimilars (RTX-B), as well as those who switched to RTX-B receiving at least one course of RTX-O.

Clinical data were extracted retrospectively through a medical chart review process. Electronic health records were evaluated based on a standard workflow agreed in preliminary meetings among all authors. The data extraction was carried out by clinicians involved in the management of SSc patients. Clinical indications for B cell depleting therapy were recorded based on organ involvement and the clinical manifestations, with a primary clinical reason for RTX initiation clearly identified for each patient. Data on RTX therapeutic regimen at the first therapeutic course and during follow-up were also collected.

For all patients, a comprehensive analysis of disease characteristics and organ involvement was available. The collected disease characteristics included: disease subset, i.e. limited cutaneous (lcSSc) or diffuse cutaneous (dcSSc) disease, autoantibody profile, disease duration (calculated from the first non-Raynaud’s symptom), extent of skin involvement according to mRSS, and presence of SSc-related organ involvements [including lung, i.e. ILD diagnosed at lung high resolution CT and/or pulmonary arterial hypertension (PAH) at right heart catheterization, primary heart involvement [15], arthritis, myositis, history of digital ulcers (DUs) or presence of active DUs at time of enrolment, gastrointestinal involvement and renal crisis].

Patients were longitudinally monitored for up to 5 years, starting from the index date of RTX introduction. The follow-up period ended at treatment discontinuation, death from any cause or the conclusion of the available follow-up, whichever occurred first.

The following outcome measures were evaluated: (i) RTX discontinuation for any reason, (ii) RTX discontinuation due to adverse events (AEs), (iii) RTX discontinuation due to treatment failure, (iv) RTX discontinuation due to AEs and treatment failure, and (v) RTX discontinuation due to clinical efficacy.

Clinical response was defined as: (i) improvement of at least 5 points or of 25% in patients with baseline mRSS >25 [16] in patients with diffuse progressive skin disease, i.e. the minimal clinically important differences (MCIDs) for the mRSS in dSSc patients [17–19]; (ii) absence of lung function deterioration defined according to OMERACT criteria as a relative FVC reduction of 10% or a simultaneous FVC relative reduction of 5% and a relative alveolar diffusion of carbon monoxide (DLCO) relative reduction of 15% [20]; (iii) clinical remission of arthritis, measured with disease activity score on 28 joints (CRP-DAS28; DAS28 < 2.6) [21]; (iv) resolution or improvement of other specific disease features, if present and considered as the main clinical indication for RTX therapy, as: active and recurrent DUs, myositis (based on clinical signs and total creatine phosphokinase serum levels) and myocarditis (based on clinical signs, high-sensitive troponin T and/or cardiac magnetic resonance findings).

Any change in immunosuppressive therapy during follow-up was recorded, including the addition or switch of conventional or biologic DMARDs, and addition of the anti-fibrotic drug nintedanib for progressive ILD. The following outcome measures after RTX discontinuation at the latest available follow-up were also recorded: disease-related deaths, any change in immunosuppressive therapy, the addition of anti-fibrotic therapy, and need for autologous haematopoietic stem cell transplantation (AHSTC) or lung transplantation.

Data on repeated RTX courses during follow-up were collected. According to clinical practice at each referral centre, mRSS was evaluated at baseline and at least every 6 months after the first RTX course during follow-up. Pulmonary function tests with FVC, total lung capacity, and DLCO before RTX treatment and during follow-up were also reviewed.

All AEs that occurred after RTX initiation were recorded, including hospitalization and severe AEs.

The study was conducted in accordance with the recommendations of the Declaration of Helsinki and was specifically approved by the local ethical committee at the coordinating centre (Comitato Etico IRCCS Ospedale San Raffaele—study code: IMMUNORADAR). The local ethical committees of all centres approved the study (Supplementary File; Supplementary Table 3, available at *Rheumatology* online). A written informed consent was obtained from each patient at each centre according to local procedures.

### Statistical analysis

Categorical variables were presented as numbers and percentages. Continuous variables were presented either as mean with standard deviation (s.d.) or as median with interquartile range (IQR), depending on the normality of the distribution as indicated by quantile–quantile plots.

The occurrence of outcomes was quantified in terms of incidence rate and 5-year cumulative incidence, with a 95% CI. The Kaplan–Meier method was used to compute the cumulative incidence in the at-risk population. A competing risk analysis was conducted to explore the clinical variables associated with RTX discontinuation for each specific cause, in comparison with alternative causes. The outcome sub-distribution hazard function was modelled using the Fine–Gray regression model. Associations were expressed as a sub-hazard ratio (sHR) with 95% CI. For the multivariate analysis, a *P*-value of <0.05 was considered statistically significant, and all tests were two-tailed. The statistical analysis was performed using RStudio, version 2023.06.1.

## Results

### Baseline evaluation and reasons for RTX initiation

We enrolled 152 SSc patients from eight centres. The cohort had a mean age of 47.3 (12.3) years, with 79.6% females and a mean disease duration of 146.9 (79.2) months. The majority of patients presented with a dSSc (77.7%) with ILD in 75.7% of cases, gastrointestinal involvement in two-thirds of cases and history of DUs in 59.9%. Overall, musculoskeletal involvement was diagnosed in the 41.4% of patients, while primary heart involvement, PAH and renal disease were only rarely present. The demographic and clinical characteristics of SSc patients are summarized in Table 1.

**Table 1.** Demographic and clinical characteristics of SSc patients

Characteristic	Value (*n* = 152)
Age, mean (s.d.), years	47.3 (12.3)
Females, *n* (%)	121 (79.6)
Disease duration, mean (s.d.), months	146.9 (79.2)
Diffuse cutaneous disease, *n* (%)	118 (77.6)
Anti-topoisomerase I, *n* (%)	96 (63.2)
Anti-centromere, *n* (%)	25 (16.4)
Anti-RNA polymerase III, *n* (%)	18 (11.8)
Others SSc-associated Abs, *n* (%)	13 (8.6)
Rheumatoid factor, *n* (%)	20 (14.1)
ACPA, *n* (%)	11 (7.9)
Overlap with rheumatoid arthritis, *n* (%)	23 (15.1)
Overlap with other CTDs, *n* (%)	13 (8.6)
Interstitial lung disease, *n* (%)^a^	115 (75.7)
Gastrointestinal involvement, *n* (%)	96 (63.2)
History of digital ulcers, *n* (%)	91 (59.9)
Active digital ulcers, *n* (%)	69 (45.4)
Musculoskeletal involvement, *n* (%)	63 (41.4)
Myositis, *n* (%)	21 (13.8)
Calcinosis, *n* (%)	26 (17.1)
Pulmonary arterial hypertension on RHC, *n* (%)	21 (13.8)
Primary heart involvement, *n* (%)	18 (11.9)
Renal involvement, *n* (%)	2 (1.4)

a ILD defined as at least 10% of interstial lung abnormalities or FVC <79%. Abs: antibodies; CTDs: connective tissue diseases; ILD: interstitial lung disease; FVC: forced vital capacity; RHC: right heart catheterization.

Notably, almost all patients (92.8%) received previous immunosuppressive therapy, primarily mycophenolate mofetil (MMF) (44.7%) or cyclophosphamide (29.6%). At the time of RTX initiation, a substantial proportion of patients (70.4%) were concomitantly receiving DMARDs, predominantly MMF (37.5%) or methotrexate (36.2%). Previous and concomitant therapies are provided in Table 2.

**Table 2.** Previous and concomitant therapies in the cohort of SSc patients treated with rituximab

Therapy	Value (*n* = 152)
Previous therapies	
Patients who underwent previous immunosuppressive therapy, *n* (%)	141 (92.8)
Mycophenolate mofetil, *n* (%)	68 (44.7)
Mycophenolate daily dose, mean (s.d.), mg	1931.0 (499.5)
Total months of MMF therapy, mean (s.d.)	45.9 (68.5)
Cyclophosphamide (total), *n* (%)	45 (29.6)
Intravenously CYC, *n* (%)	26 (17.1)
Cyclophosphamide cumulative dose, mean (s.d.), g	11.0 (7.3)
Azathioprine or methotrexate, *n* (%)	19 (12.5)
Other bDMARDs, *n* (%)	9 (5.9)
Concomitant therapies at first RTX course	
Corticosteroids, *n* (%)	113 (74.3)
Steroids daily dose, mean (s.d.), mg	4.9 (4.8)
Higher steroids (>7.5 mg/day prednisone), *n* (%)	15 (9.8)
Concomitant DMARDs, *n* (%)	117 (77.0)
Methotrexate, *n* (%)	55 (36.2)
Mycophenolate mofetil, *n* (%)	57 (37.5)
Hydroxychloroquine, *n* (%)	19 (12.5)
Other DMARDs, *n* (%)	5 (3.3)
Concomitant therapy with nintedanib, *n* (%)	11 (7.2)
Calcium channel blockers, *n* (%)	95 (62.5)
Acetylsalicylic acid, *n* (%)	74 (48.7)
Intravenous iloprost, *n* (%)	104 (68.4)
Proton pump inhibitors, *n* (%)	130 (85.5)
ERAs, *n* (%)	48 (31.6)
PDE5i, *n* (%)	18 (11.8)

CYC: cyclophosphamide; ERAs: endothelin receptor antagonists; MMF: mycophenolate mofetil; PDE5i: phosphodiesterase 5 inhibitors; RTX: rituximab.

### RTX regimens and follow-up

The main reason for initiating RTX was ILD progression in 38.8% of patients, worsening skin fibrosis in 36.8%, and presence of arthritis in 13.8%. RTX was prescribed for multiple indications in 41 cases (27.0%), with 21 patients (13.8%) receiving RTX for concomitant skin and lung disease progression. The majority of patients (71) received RTX-O (46.7%) and later switched to RTX-B. In contrast, 61 patients (40.1%) received RTX-B from the first therapeutic course, and only 20 patients (13.2%) remained on RTX-O. Almost all patients (98.7%) were treated with a dose of RTX 1000 mg repeated after 15 days, while only two patients received RTX 500 mg every 7 days for 4 weeks.

Patients were monitored for up to 5 years, with the median (IQR) follow-up time being 3.3 (1.7–5.0) years.

During this period, 138 patients (90.8%) underwent more than one RTX course (median of 4 [3–8] courses). In 113 cases (81.9%), RTX was repeated at 6 months, while in 25 cases (18.1%) the timing of subsequent courses was clinically driven, i.e. administered on demand.

Most patients who received at least a second course of RTX after 6 months continued with the same therapeutic regimen (62.5%). However, the RTX dose was reduced to a single 1000 mg dose in 34 cases (22.4%) and to a single 500 mg dose in six cases (3.5%), due to the satisfactory clinical response. Changes in concomitant therapies after the first RTX course and during the follow-up period are detailed in Supplementary Table 1, available at *Rheumatology* online.

### RTX retention rate and causes of discontinuation

**Figure 1.**
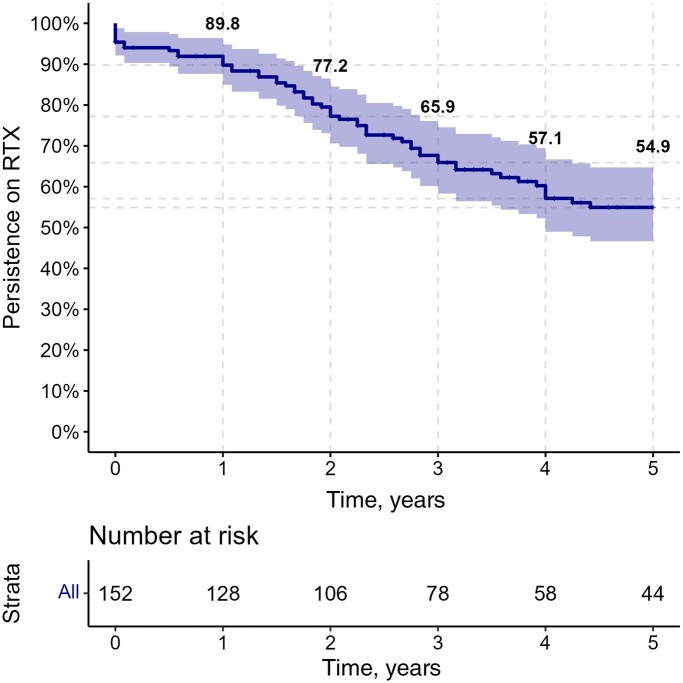
Rituximab (RTX) retention rate.

The retention rate of RTX over five years of treatment is illustrated in the Kaplan-Meier curve presented in Fig. 1. The RTX retention rates (95% CI) at 1, 2, 3, 4, and 5 years were 89.8% (85.0, 94.8%), 77.2% (70.6, 84.5%), 65.9% (58.3, 74.5%), 57.1% (49.0, 66.7%) and 54.9% (46.6, 64.7%), respectively (Supplementary File, available at *Rheumatology* online).

**Figure 2.**
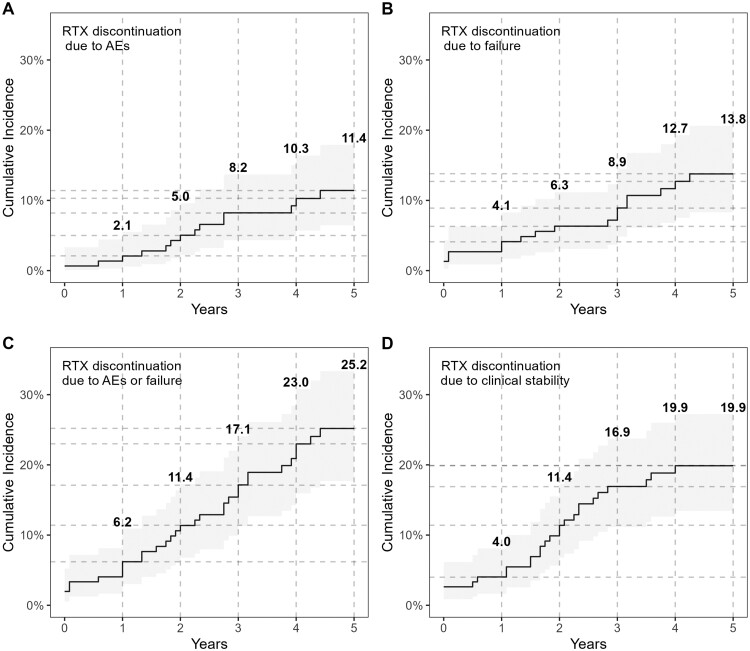
Rituximab (RTX) discontinuation event rates and 5-year cumulative incidence. There were fifty-one discontinuation events: clinical response (26), treatment failure (17), infusion reaction (7), infections (4), cardiovascular events (2), cytopenia (1). AEs: adverse events

The cumulative incidences of RTX discontinuation for specific reasons are depicted in Fig. 2. Clinical response was the most common cause of RTX discontinuation occurring at the rate of 5.7 (95% CI: 3.7–8.4) per 100 patient-years and was associated with a shorter disease duration (sHR 0.8; 95% CI: 0.7, 0.9), anti-topoisomerase-I negativity (sHR 0.4; 95% CI: 0.2, 0.9), history of DUs (sHR 2.6; 95% CI: 1.1, 6.2) and absence of arthritis (sHR 0.3; 95% CI: 0.1, 0.8) (Supplementary Fig. 1, available at *Rheumatology* online).

Both, at 6 months and at final follow-up, skin score was significantly improved in the entire cohort [mRSS: 11.9 (8.0) and 8.3 (7.4) *vs baseline*: 16.5 (9.6)] and in particular in dcSSc patients characterized by skin disease progression [mRSS: 15.6 (9.6) and 9.2 (7.9) *vs* 21.0 (10.8)] (*P* < 0.001 for both comparisons). At 6 months and at final follow-up, mean predicted (%) FVC and DLCO remained stable in the entire cohort [FVC: 87.3 (21.5) and 87.0 (23.5) *vs* 87.4 (19.8); DLCO: 65.2 (20.9) and 61.1 (19.6) *vs* 62.1 (19.7)] and in ILD patients [FVC: 84.7 (19.2) and 82.5 (21.6) *vs* 81.6 (17.8); DLCO: 64.5 (19.0) and 57.9 (18.5) *vs* 60.6 (18.7)] (all not statistically significant).

Considering only the 26 patients who discontinued RTX for clinical response, both at 6 months and at final follow-up, skin score was significantly improved [mRSS: 14.0 (10.3) and 8.2 (7.1) *vs baseline*: 19.7 (9.3)] (*P* < 0.001 for both comparisons). Similarly, mean predicted (%) FVC and DLCO remained stable at 6 months and at final follow-up [FVC: 82.3 (21.5) and 82.1 (21.1) *vs* baseline: 86.3 (19.9); DLCO: 63.6 (20.8) and 62.3 (21.5) *vs* baseline: 64.0 (22.1)] (all not statistically significant).

Considering the 26 patients who discontinued RTX due to clinical response, the majority of them were already treated with DMARDs (92.3%) before RTX, and 13 of them (50%) were concomitantly treated with DMARDs.

Treatment failure was the second cause of RTX discontinuation occurring at 3.7 (95% CI: 2.2, 6.0) per 100 patient-years, and was associated with anti-centromere antibody positivity (sHR 2.8; 95% CI: 1.1, 7.4) and anti-topoisomerase-I negativity (sHR 0.2; 95% CI: 0.1, 0.6) (Supplementary Fig. 2, available at *Rheumatology* online). To date, however, considering the seven patients with anti-centromere positivity who discontinued RTX for treatment failure, all of them had a severe disease, refractory to previous DMARDs and that subsequently required anti-fibrotic and/or biologic therapy or AHSCT; moreover, in four of these cases RTX was mainly started for refractory digital ulcers or calcinosis.

Considering the 20 patients who discontinued RTX due to treatment failure, all of them were already treated with DMARDs before RTX, and 15 of them (75%) were concomitantly treated with DMARDs, mainly mycophenolate. After RTX discontinuation, at latest available follow-up [median 39 (23.25–51.50)], 16 out of 20 (80%) patients needed further therapy: tocilizumab was initiated in six patients (30%) and nintedanib in four (20%); three patients, moreover, underwent AHSTC, and one patient each received cyclophosphamide, intravenous immunoglobulins, or Janus kinase inhibitors.

Overall, 42 AEs were recorded, mild infection being the most frequent (19.1%), followed by hypersensitivity reactions (15.1%) and mild leukopenia (15.1%). However, in only 14 cases AEs led to drug discontinuation (9.2%): seven infusion reactions, four severe infections, two cardiovascular events (including one case of myocarditis after SARS-CoV-2 infection and one severe persistent hypotension) and one cytopenia (Supplemental Table 2, available at *Rheumatology* online). The occurrence of AEs was the less common cause of RTX discontinuation at a rate of 3.1 (95% CI: 1.7, 5.2) per 100 patient-years, associated with limited cutaneous subset (sHR 3.4; 95% CI: 1.2, 9.7) and previous MMF treatment (sHR 4.5; 95% CI: 1.2, 16.3) (Supplementary Fig. 3, available at *Rheumatology* online). AEs occurred after a median time of 6 (1.0–24.0) months after RTX initiation with high variability, with some patients presenting infusion reactions rapidly emerging after the first RTX cycle (18 AEs occurring in the first 3 months after RTX), and patients presenting with late occurring AEs (18 AEs occurring >6 months after RTX cycle).

Considering the 14 patients who discontinued RTX due to AEs, all of them were already treated with DMARDs before RTX, and 12 of them (85.7%) were concomitantly treated with DMARDs, mainly mycophenolate.

Changes in therapy after RTX discontinuation due to clinical response, clinical failure and AEs are reported in Supplementary File, available at *Rheumatology* online.

The retention rate was not associated with the clinical indication for RTX therapy, nor with the autoantibody profile or the previous and concomitant therapies. Furthermore, the retention rate was not associated with the therapeutic regimen at first RTX course and during follow-up, the use of RTX-O *vs* RTX-B, or the decision to repeat RTX courses every 6 months or as clinically needed (not statistically significant for all the comparisons).

## Discussion

Our long-term, real-life data suggest that RTX is a safe treatment in SSc patients, with limited impact from AEs on treatment retention. Indeed, AEs emerged as the least frequent cause of RTX discontinuation over a 5-year follow-up period. Overall, AEs leading to drug discontinuation were recorded only in 14 patients (9.2%), predominantly due to infusion reactions.

Regarding treatment efficacy, RTX was shown not only to be effective in the whole population, but its administration was able to counteract disease activity especially in SSc patients with early disease and a more favourable antibody profile. Indeed, one of the most striking results of our study was the observation that clinical response emerged as the primary reason for RTX discontinuation, associated with shorter disease duration and anti-topoisomerase I negativity. However, the association of RTX discontinuation with clinical response anti-topoisomerase-I negativity needs to be taken with caution. The disease specific autoantibody profile in two-thirds of patients with anti-topoisomerase and anti-centromere negativity who discontinued RTX for clinical response, indeed, was not clarified.

The hypothesis that RTX could be especially effective in the early stage of the disease has been previously suggested [4, 5, 22, 23]. The clinical usefulness of RTX on skin and lung involvement in patients with <3 years of disease duration, indeed, emerged in two studies assessing the role of B cell depleting therapy in SSc [5, 22]. In early SSc, an interplay between innate immunity, the pro-inflammatory/pro-fibrotic cytokine milieu and the adaptive immunity involving T cell and B cell dysregulation has been described [24, 25]. In fact, B cell infiltration in the skin is present in early diffuse cutaneous disease and correlates with fibrosis progression [26]. This evidence provides a biological rationale for using RTX in the early stages of the disease to slow down progression to fibrosis. However, the efficacy of RTX must be rigorously investigated through randomized trials, because the B cell depleting therapy may be an early intervention to modify disease progression by interfering with the immune-inflammatory process leading to fibrosis.

Recently, nintedanib has been shown to reduce the annual rate of decline of FVC in SSc ILD in a double-blind placebo-controlled study [3], leading to approval of its use for the treatment of ILD in SSc. Unfortunately, this treatment did not demonstrate a significant effect on skin involvement in the SENSCIS trial [3]. Similarly, the primary end point for skin improvement was not met in the FaSScinate trial with tocilizumab [6], although it did show beneficial effects on lung function. Finally, no medications have shown definitive benefits for other disease manifestations, including musculoskeletal, cardiac and gastrointestinal involvement.

Although our study was not specifically designed to assess the efficacy of RTX in specific disease manifestations of SSc, the RTX-related improvement of skin fibrosis and stabilization of lung function over long term use was confirmed. This aligns with findings from previous studies [4, 5, 22, 23] and has been recently supported by the data from the EUSTAR registry [9, 27]. Comparable results have been also obtained with RTX biosimilars [11], and these findings are further backed by meta-analyses [10, 28, 29] and the recent DESIRES trial [7]. However, even considering the DESIRES trial extension study [12], it only reported the efficacy and safety of RTX up to 48 weeks, leaving a gap in long-term data. Our study, therefore, provides the longest follow-up available on RTX in SSc patients, offering insights on real-life, long-term efficacy and safety of the drug. Interestingly, regarding patients who discontinued RTX for clinical response, after drug discontinuation the majority of them remained stable during a long follow-up even without any DMARDs as maintenance therapy, or by continuing the same DMARD as before RTX.

The likelihood of discontinuing RTX due to clinical response was not influenced by the therapeutic regimen at the initial RTX course and during follow-up, nor by the decision to repeat RTX courses every 6 months or as clinically needed. Remarkably, 10% of our patients responded to one cycle, suggesting a specific disease phenotype or a window of opportunity where B cell blockade is a critical intervention in the disease’s progression.

Considering the long period of observation of our study, however, we have to consider that our data mirror probably also the modification in use of RTX in SSc patients over recent years.

Treatment failure was the second most common reason for discontinuing RTX, associated with anti-centromere antibody positivity and anti-topoisomerase-I negativity. This association is not surprising, considering that skin and lung progression—the two primary indications for RTX—are more frequent in patients with anti-topoisomerase-I positivity [1, 2], and studies on RTX efficacy in SSc have mainly reported a skin improvement and a lung function stabilization [4, 5, 22, 23]. Therefore, we can hypothesize that anti-centromere positive patients might have a lower chance to respond to RTX therapy, because the clinical phenotype of these patients was different and more heterogeneous. In fact, in our cohort, anti-centromere positivity was linked to disease manifestations where RTX efficacy is less established, such as DUs and calcinosis, and with clinical features suggesting a refractory disease. Interestingly, though, the percentage of patients concomitantly treated with conventional DMARDs among those who discontinued RTX due to treatment failure was higher compared with those who discontinued RTX for clinical response (75% *vs* 50%), thus indirectly suggesting that patients who discontinued RTX due to treatment failure had a more severe disease. No safety concerns emerged in patients treated with a combined immunosuppressive regimen.

Our study has some limitations: firstly, its retrospective nature inherently restricts the possibility of drawing definitive conclusions, and, secondly, the clinical indications for initiating RTX and for repeated courses in patients not prescribed with a fixed retreatment scheme were largely based on physicians’ judgment. The study was conducted in national SSc referral centres, ensuring high expertise in disease management and homogeneity in treatment decisions. However, the significant lack of data on anti-topoisomerase-I negativity and anti-centromere negativity did not allow identification of any association between autoantibody profile and the clinical response to RTX. Eventually, the data were collected over >13 years during which diagnostic approaches, monitoring strategies, and therapeutic management of SSc patients evolved, influencing the clinical indications for RTX therapy and for RTX re-treatment, as well as the definition of clinical response.

In conclusion, our real-life long-term data indicate that RTX is a safe treatment for SSc patients with AEs having a limited impact on treatment persistence, allowing a long lasting treatment. Additionally, our data confirm the efficacy of RTX, which is seen especially in early SSc. In the near future, the identification of specific disease features and autoantibody profile associated with a response to RTX will be useful in the management of SSc patients.

## Supplementary Material

keae280_Supplementary_Data

## Data Availability

The data that support the findings of this study are available on request from the corresponding author (G.L.D.).
