# Supplementary material for: Rituximab retention rate in systemic sclerosis: a long term real-life multicentre study
Source: Rheumatology (Oxford). 2024 May 15;64(3):1284–91. doi: 10.1093/rheumatology/keae280 (PMC11879284; doi:10.1093/rheumatology/keae280)
Supplement: keae280_Supplementary_Data [file keae280_supplementary_data.zip › keae280_Supplementary_Data/rhe-24-0502-File004.docx]

**RTX retention rate, causes of discontinuation and follow-up after discontinuation**

Eighty-seven patients (57.3%) reached a 3-year follow-up, 62 patients (40.8%) a 4-year follow-up and 45 patients (29.6%) a 5-year follow-up.

Considering the 26 patients who discontinued rituximab (RTX) for clinical response, after RTX discontinuation, at latest available follow-up (median 48.0 [25.25 - 73.50] months), 18 patients (69.2%) continued to be treated as before RTX, chiefly without any immunosuppressive therapy in 11 cases (61.1%), or the previously received DMARDs in the remaining 7 cases (38.9%); conversely, 5 patients (19.2%) started nintedanib for progressive interstitial lung disease (ILD) (after a median time of 22 [17.0 – 47.0] months) and 2 patients (7.7%) received tocilizumab (after 34 months and 6 months, respectively).

Considering, the 20 patients who discontinued RTX due to treatment failure, after RTX discontinuation, at latest available follow-up (median 39 [23.25 - 51.50]), 80% of patients needed further therapies: tocilizumab was initiated in 6 patients (30%) and nintedanib in 4 (20%); 3 patients, moreover, underwent AHSTC, and 1 patient each received cyclophosphamide, intravenous immunoglobulins, or Janus Kinase (JAK)-inhibitors*.*

Considering, finally, the 14 patients who discontinued RTX due to adverse events, instead, after RTX discontinuation, at latest available follow-up (median 75 [48.0 – 88.0]), the majority of patients (78.6%) continued therapy with conventional disease modifying anti-rheumatic drugs (DMARDs) (mainly mycophenolate and rituximab), while tocilizumab, nintedanib and autologous hematopoietic stem cells transplantation (AHSTC) were needed in 1 patient each.

**Supplemental Table 1. Changes in concomitant therapies after first rituximab course and during follow-up.**

| ***Changes in therapies after first RTX course*** | **N= 152** |
| --- | --- |
| Steroid dose reduction after RTX initiation, n (%) | 56 (36.8) |
| Steroids suspension after RTX initiation, n (%) | 25 (16.5) |
| Not modified DMARDs during RTX, n (%) | 108 (71.1) |
| Suspension of DMARDs during RTX, n (%) | 25 (16.4) |
| Switch to other DMARD during RTX, n (%) | 16 (10.5) |
| Addition of DMARD during RTX, n (%) | 23 (15.1) |
|  |  |
| **Changes in therapies during follow-up** |  |
| Switch to tocilizumab, n (%) | 3 (2.0) |
| Addition of DMARDs due to progression or not complete response, n (%) | 52 (34.2) |
| MMF addition, n (%) | 22 (42.3)* |
| Switch to different DMARD, n (%) | 7 (13.5) |
| IVIG addition, n (%) | 5 (9.6) |
| CYC initiation, n (%) | 4 (7.7) |
| Nintedanib addition, n (%) | 7 (13.5) |

*N= number; RTX= rituximab; DMARDs= disease modifying anti-rheumatic drugs; n= number; MMF= mycophenolate mofetil; IVIG= intravenous immunoglobulins; CYC= cyclophosphamide.*

********calculated among the 52 patients who needed a DMARDs addition.*

**Supplemental Table 2. Adverse events during rituximab therapy.**

| **Adverse events** | **N=152** |
| --- | --- |
| Total adverse events, n (%) | 42 (27.6) |
| Hypersensitivity reactions, n (%) | 23 (15.1) |
| Systemic inflammatory reaction, n (%) | 3 (2.0) |
| Infections, n (%) | 29 (19.1) |
| Severe infection, n (%) | 16 (10.5) |
| Re-activation of chronic infections, n (%) | 2 (1.3) |
| Leukopenia, n (%) | 10 (15.2) |
| Severe leukopenia, n (%) | 2 (1.3) |
| Hypogammaglobulinemia | 11 (7.2) |
| Severe hypogammaglobulinemia, n (%) | 1 (0.7) |

**Supplementary Table 3: Local Ethical Committee approval information**

| **Site of patient enrolment** | **City** | **Ethical Authority** |
| --- | --- | --- |
| Unit of Immunology, Rheumatology, Allergy and Rare Diseases, IRCCS San Raffaele Hospital | Milan  (Italy) | Comitato Etico IRCCS Ospedale San Raffaele, code: IMMUNORADAR |
| Unit of Rheumatology, Catholic University of the Sacred Heart, Fondazione Policlinico Universitario A. Gemelli IRCCS | Rome  (Italy) | Comitato Etico Policlinico A. Gemelli 0002461/23 |
| Scleroderma Clinic, ASST Gaetano Pini-CTO, Milan, Italy | Milan  (Italy) | Comitato Etico ASST Gaetano Pini – ID number: 339 – study number: 6549 |
| Rheumatology Unit, Department of Emergency and Organs Transplantation, University of Bari | Bari (Italy) | Comitato Etico AOUC Policlinico di Bari, protocol n. 5351/2017 |
| Rheumatology Unit, Padova University Hospital | Padua (Italy) | Comitato Etico Azienda Ospedaliera di Padova 5505/AO/22 |
| Scleroderma Unit, UOC Reumatology and Clinical Immunology, ASST Spedali Civili, Brescia | Brescia (Italy) | Comitato Etico ASST Spedali Civili – code: NP2366 |
| Department of Rheumatology, University of Modena and Reggio Emilia | Modena  (Italy) | Comitato Etico AOU Modena – code: SCLERORER 3826 |
| Rheumatology and Clinical Immunology, IRCCS Humanitas Research Hospital | Rozzano (Italy) | Comitato Etico Humanitas Research Hospital, code: 0831 |

**Supplemental Figure 1 Legend. Rituximab discontinuation due to clinical response.**

Clinical response was the most common cause of RTX discontinuation occurring at the rate of 5.7 (3.7-8.4) per 100 patient-year and was associated with a shorter disease duration (sHR 0.8, 95% CI 0.7-0.9), anti-topoisomerase-I negativity (sHR 0.4, 95% CI0.2-0.9), history of DUs (sHR 2.6, 95% CI 1.1-6.2), and absence of arthritis (sHR 0.3, 95% CI (0.1-0.8).

*ACA= anti-centromere; mRSS= modified Rodnan skin score; ILD= interstitial lung disease; HRCT= lung high-resolution computed tomography; FVC= forced vital capacity, expressed ad % of predicted values; DLCO= diffusing capacity for carbon monoxide, expressed ad % of predicted values; CYC= cyclophosphamide; MMF= mycophenolate mofetil; MTX= methotrexate; CS= corticosteroids.*

**Supplemental Figure 2 Legend. Rituximab discontinuation due to treatment failure.**

Treatment failure was the second cause of RTX discontinuation occurring at 3.7 (95% CI 2.2-6.0) per 100 patient-year , and was associated with anti-centromere antibody positivity (sHR 2.8, 95% CI1.1-7.4) and anti-Scl70 negativity (sHR 0.2, 95% CI 0.1-0.6).

*ACA= anti-centromere; mRSS= modified Rodnan skin score; ILD= interstitial lung disease; HRCT= lung high-resolution computed tomography; FVC= forced vital capacity, expressed ad % of predicted values; DLCO= diffusing capacity for carbon monoxide, expressed ad % of predicted values; CYC= cyclophosphamide; MMF= mycophenolate mofetil; MTX= methotrexate; CS= corticosteroids.*

**Supplemental Figure 3. Rituximab discontinuation due to adverse events.**

The occurrence of AEs was the less common cause of RTX discontinuation at a rate of 3.1 (95% CI 1.7-5.2) per 100 patient-year, associated with limited cutaneous subset (sHR 3.4, 95% CI 1.2-9.7) and previous MMF treatment (sHR 4.5, 95% CI 1.2-16.3).

*ACA= anti-centromere; mRSS= modified Rodnan skin score; ILD= interstitial lung disease; HRCT= lung high-resolution computed tomography; FVC= forced vital capacity, expressed ad % of predicted values; DLCO= diffusing capacity for carbon monoxide, expressed ad % of predicted values; CYC= cyclophosphamide; MMF= mycophenolate mofetil; MTX= methotrexate; CS= corticosteroids.*
