# Supplementary figures and images for: Rituximab retention rate in systemic sclerosis: a long term real-life multicentre study
Source: Rheumatology (Oxford). 2024 May 15;64(3):1284–91. doi: 10.1093/rheumatology/keae280 (PMC11879284; doi:10.1093/rheumatology/keae280)

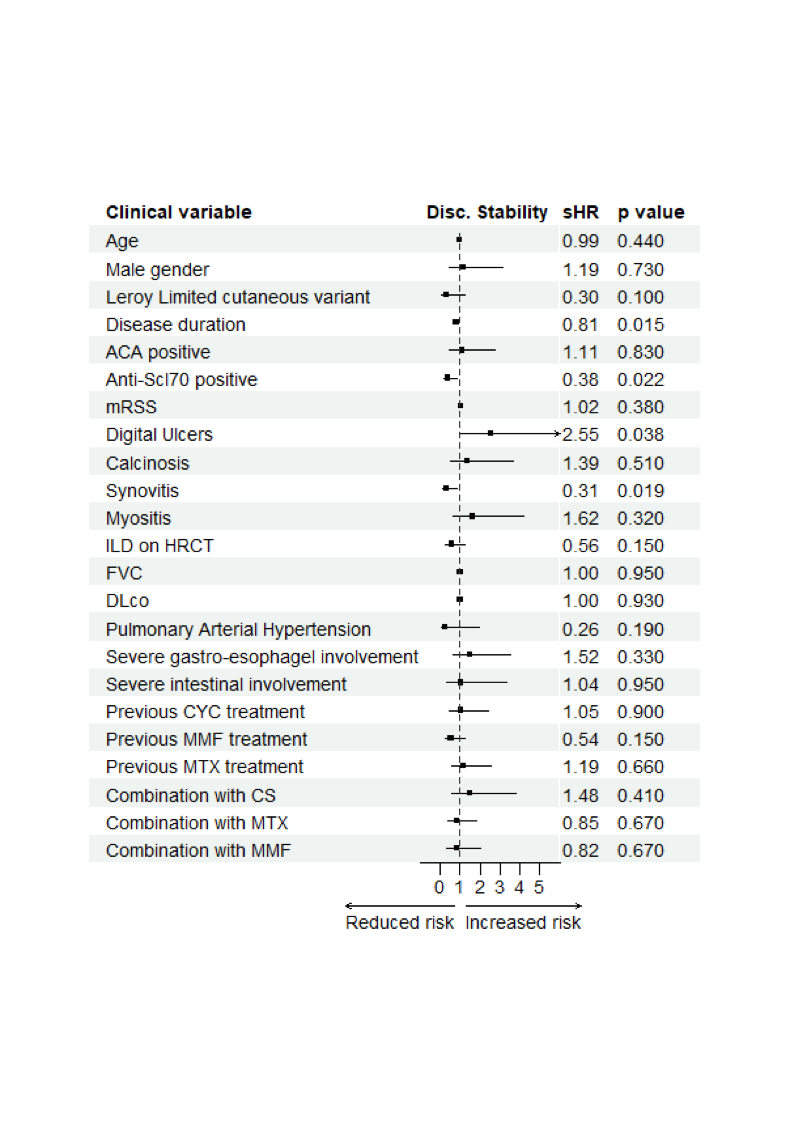

Supplement: keae280_Supplementary_Data [file keae280_supplementary_data.zip › keae280_Supplementary_Data/rhe-24-0502-File005.png]

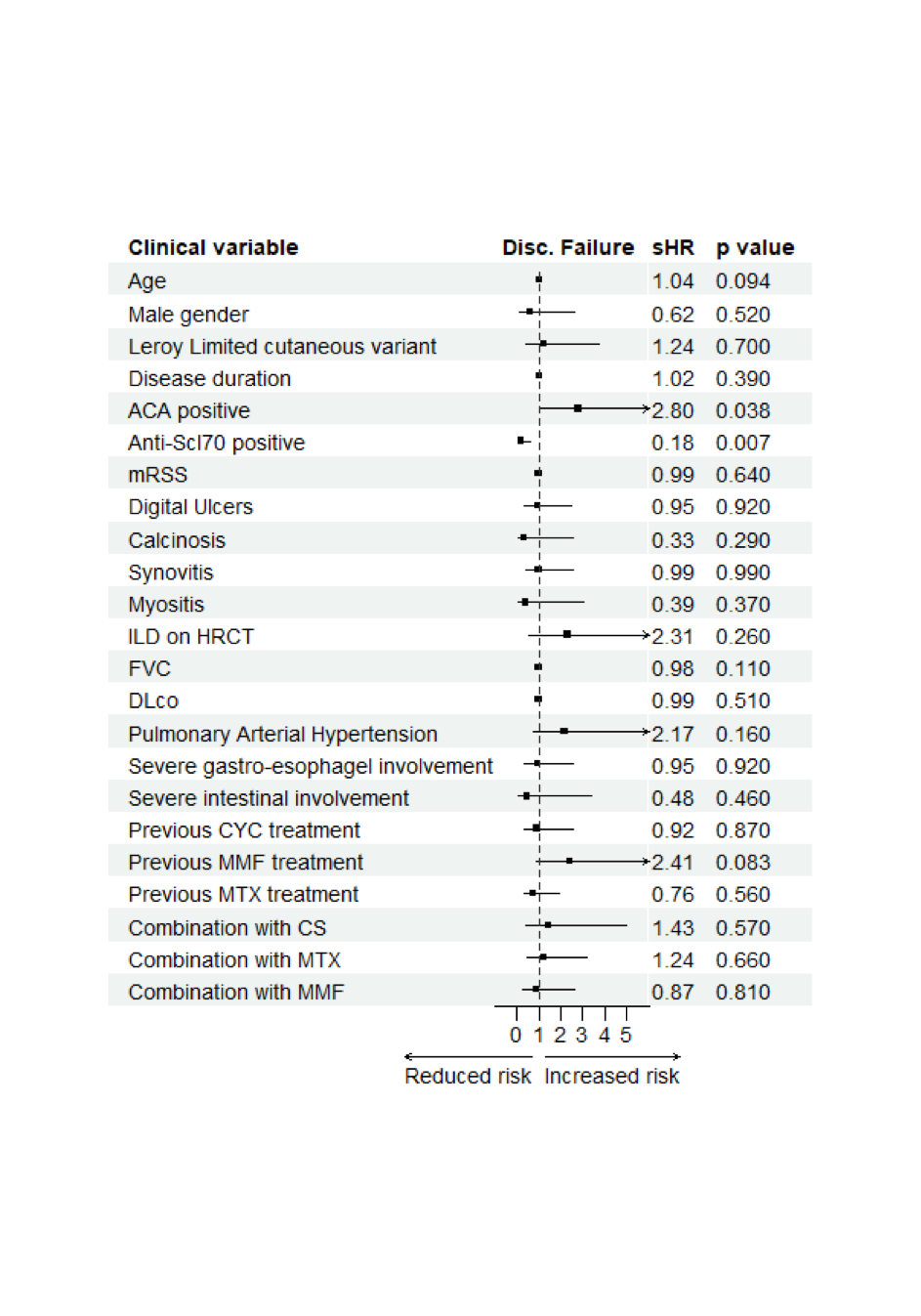

Supplement: keae280_Supplementary_Data [file keae280_supplementary_data.zip › keae280_Supplementary_Data/rhe-24-0502-File006.tiff]

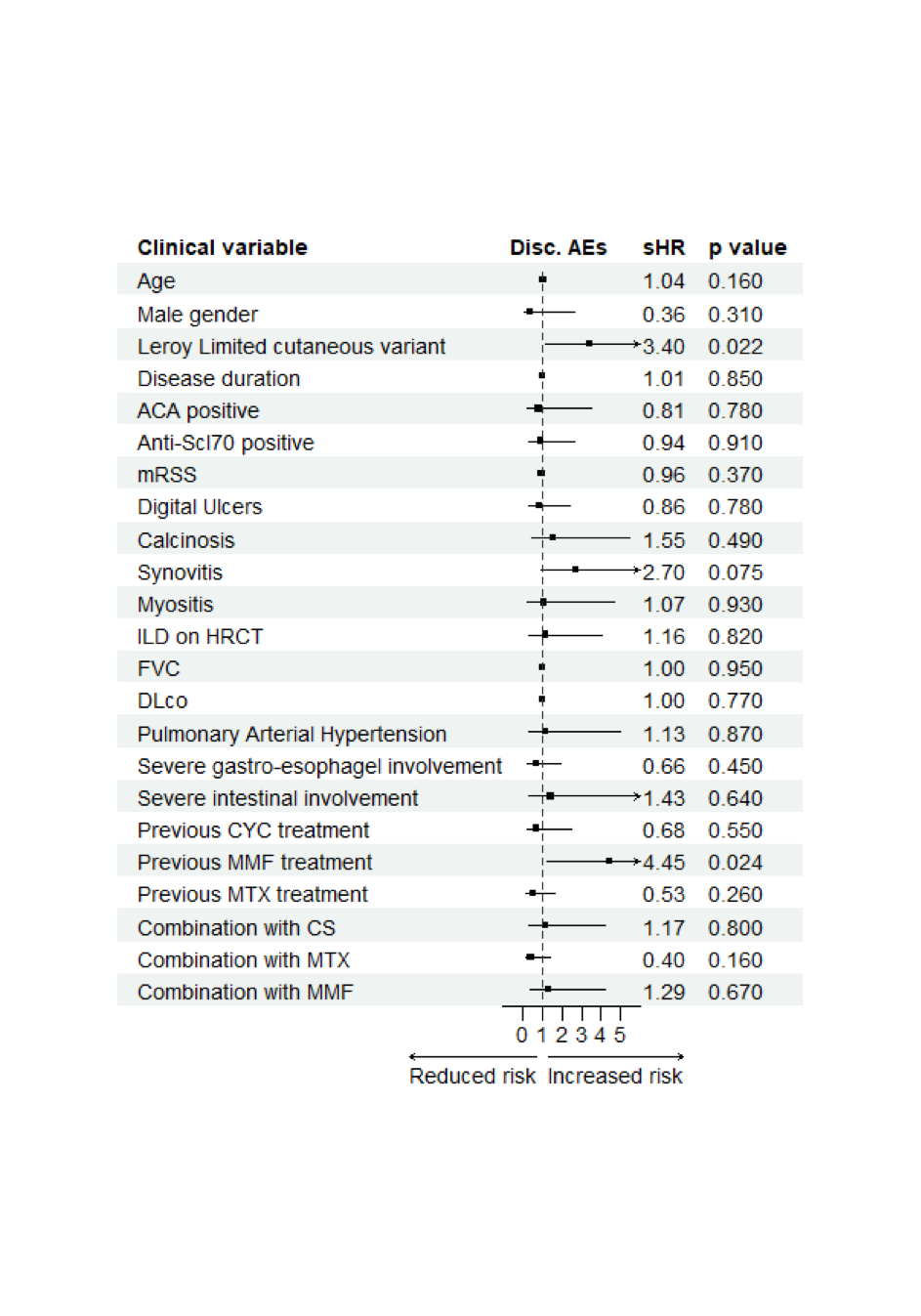

Supplement: keae280_Supplementary_Data [file keae280_supplementary_data.zip › keae280_Supplementary_Data/rhe-24-0502-File007.tiff]
